# Evidence-Based Prevention of De Novo GERD after Bariatric Surgery: Comparing Human and AI Inference

**DOI:** 10.1007/s11695-026-08511-w

**Published:** 2026-02-10

**Authors:** Mohammad Kermansaravi, Salvatore Tolone, Daniel Gero, Shahab Shahabi Shahmiri, Amir Hossein Davarpanah Jahazi, Panagiotis Lainas, Sonja Chiappetta

**Affiliations:** 1 Department of Surgery, Minimally Invasive Surgery Research Center, Division of Minimally Invasive and Bariatric Surgery, Hazrat-E Fatemeh Hospital, Iran University of Medical Sciences, Tehran, Iran; 2 University of Campania “Luigi Vanvitelli”, Naples, Italy; 3 Department of Surgery and Transplantation, University Hospital of Zurich, Zurich, Switzerland; 4 Department of Metabolic & Bariatric Surgery, Metropolitan Hospital, Athens, Greece; 5 Division of Surgery, School of Medicine, European University Cyprus, Nicosia, Cyprus; 6 Obesity and Metabolic Surgery Unit, Ospedale Evangelico Betania, Naples, Italy

## Introduction

With the global rise in obesity rates, metabolic and bariatric surgery (MBS) has become the primary treatment modality for managing obesity and its associated complications. Gastroesophageal reflux disease (GERD), a common complication of obesity primarily caused by increased intra-abdominal pressure and impaired lower esophageal sphincter function, often shows improvement following MBS. However, paradoxically, MBS can also lead to the development of de novo GERD in some patients [1]. Although de novo GERD can be an inherent consequence of MBS procedures, particularly those that are naturally refluxogenic [2, 3], the risk is not solely procedure-dependent. Technical refinements during surgery can play a crucial role in preventing or reducing the incidence of de novo GERD. Furthermore, some patients may be inherently more susceptible to developing GERD postoperatively, highlighting the importance of careful patient selection [4, 5]. Standardization of preoperative evaluation, diagnostic definitions, and risk assessment protocols is also essential to improve preventive strategies [6, 7]. Despite its clinical relevance, there is currently no established guideline or consensus on how best to prevent de novo GERD following MBS. With the advent of artificial intelligence (AI), particularly large language models (LLMs), clinicians now have powerful tools to access, interpret, and apply vast amounts of clinical data [8]. These technologies can support surgeons in selecting suitable candidates, selecting the optimal surgical procedure, and refining technical details to minimize the risk of de novo GERD. This study aims to provide an evidence-based framework for the diagnosis and prevention of de novo GERD after MBS, while also comparing human clinical reasoning with AI-driven inference in this context.

## Methods

Based on a structured evidence search, the team (seven bariatric surgeons) formulated 25 declarative clinical statements across four domains: preoperative assessment, patient selection, procedure selection, and surgical technical considerations. Each statement was written as a binary, testable proposition (answerable with “yes” or “no”) and linked to a predefined level of evidence as shown in Table 1. The draft statements were iteratively refined by the team through extensive discussion to ensure clinical relevance, unambiguous wording, and direct traceability to the underlying studies or expert recommendations.

**Table 1:** Levels of evidence in clinical research

Level	Type of Evidence	Description
Level I	High-quality randomized controlled trials (RCTs), or systematic reviews/meta-analyses of RCTs	Strongest level of evidence; low risk of bias, large sample size, consistent results
Level II	Lesser-quality RCTs, prospective comparative studies	RCTs with some methodological limitations (e.g., small sample size, lack of blinding)
Level III	Retrospective cohort studies, case-control studies	Observational studies with a control group, but a higher risk of bias
Level IV	Case series, poor-quality cohort, and case-control studies	Descriptive studies without control groups or with significant design flaws
Level V	Expert opinion, case reports, bench research, and theoretical reasoning	No direct clinical evidence; based on clinical experience or indirect evidence

Four publicly accessible LLM-based systems were evaluated: ChatGPT-4o (OpenAI, gpt-4o-2025-03-27 version active on the test date), Gemini (Google), Consensus.app (an LLM‑based scientific literature search and synthesis engine), and Grok 3 (xAI). All queries were submitted through their respective web interfaces on 01 May 2025, using the production versions available to users on that date, with no additional fine-tuning or custom settings applied. For the LLMs evaluation, each of the 25 statements was converted into a standardized prompt using the identical wording across all LLMs. The generic instruction was: “Please answer the following clinical statement with ‘Yes’ or ‘No’ only, based on the current evidence in the medical literature”, followed by the statement text. No additional examples, context, or follow‑up clarifications were provided, and the models were not allowed to browse or retrieve external documents beyond their default capabilities on the test date.

To reduce potential learning effects and cross-contamination between prompts, each statement was entered in a separate new conversation or query window for each model. For all systems, the default generation parameters (including temperature and randomness) provided by the interface were used, and no manual adjustment of these settings was made. Each statement was submitted once per model, and the first complete answer was recorded for analysis without resubmission or manual editing.

### Outcome Definition and Analysis

The primary outcome was agreement between each LLM’s answer and the evidence-based expert response for each statement. For every statement, the expert reference answer (‘Yes’ or ‘No’) was determined a priori by two bariatric surgeons with reference to the underlying literature and the assigned level of evidence from Table 1 as the benchmark; disagreements were resolved by discussion. An LLM response was classified as ‘aligned’ if its final answer matched the reference Yes/No response, and ‘not aligned’ if it differed. For each model, the proportion of aligned responses across the 25 statements was calculated and reported as percentage agreement with the expert standard (Table 2).

**Table 2:** Summary of statements and comparative analysis of LLM-Generated responses versus evidence-based expert responses

Statement	Evidence-Based Expert Response	Level of Evidence	ChatGPT 4 o	Gemini	Consensus.app	Grok 3
Preoperative Assessment						
1.Can the surgeons rely only on GERD symptoms for definitive diagnosis of GERD before MBS?	**No**	**Level II**	NO	NO	NO (100%)	NO
2. Is the current definition of GERD based only on symptoms?	**No**	**Level IV**	NO	NO	YES (80%) NO (20%)	NO
3. Are Upper Endoscopy and pH-monitoring necessary testing for GERD diagnosis before MBS?	**Yes**	**Level II**,** III**,** and IV**	YES	NO	YES (100%)	YES
4. Is Esophageal High-Resolution Manometry mandatory for GERD diagnosis before MBS?	**No (yes only in case of non-obstructive dysphagia)**	**Level III**	NO	NO	NO (100%)	NO
5. Are normative data for instrumental testing the same for the population with obesity?	**No**	**Level III**	NO	NO	NO	NO
Patient Selection						
6. Is a risk assessment for de novo GERD or worsening of existing GERD following MBS necessary during the preoperative evaluation for MBS?	**Yes**	**Level II**	YES	YES	YES	YES
7. Can the American Foregut Society (AFS) classification serve as a supplementary tool in identifying patients at risk for GERD following MBS?	**Yes**	**Level II**	YES	YES	YES	YES
8. Do patients with significant preoperative GERD have a higher risk for future GERD-related sleeve complications?	**Yes**	**Level II**	YES	YES	YES (100%)	YES
9. Is it reasonable to perform SG in patients who are well informed of the risk of worsening GERD requiring additional surgical interventions?	**Yes**	**Level II**	YES	YES	YES	YES
Procedure Selection						
10. Should SG be contraindicated in patients with silent GERD + large hiatal hernia?	**No**	**Level II**	YES	YES	NO (83%) YES (17%)	YES
11. Is RYGB superior to SG for silent GERD patients, even without erosive esophagitis?	**Yes**	**Level I**	YES	YES	YES (64%) Possibly (18%) NO (18%)	YES
12. Is RYGB superior to SG in against the occurrence of de novo GERD?	**Yes**	**Level I**	YES	YES	YES (82%) NO (18%)	YES
13. Does SG have a higher risk for developing Barrett’s Esophagus and reflux esophagitis compared to RYGB?	**Yes**	**Level I**,** II**	YES	YES	YES (64%) Mixed (7%) NO (29%)	YES
14. Is RYGB superior to OAGB against the occurrence of de novo GERD?	**Yes**	**Level I**	YES	YES	YES (50%) NO (50%)	YES
15. Does OAGB increase GERD risk compared to RYGB in silent GERD patients?	**Yes**	**Level I**	YES	YES	NO (75%) YES (25%)	YES
Surgical Technical Considerations						
16. Can a small hiatal hernia (less than 2–4 cm) be left unrepaired during SG without increasing the risk of de novo GERD?	**Yes**	**Level I/II/III**	NO	YES	YES	YES
17. Does repairing a medium or large hiatal hernia during SG reduce the incidence of de novo GERD?	**Yes**	**Level I/II/III**	YES	YES	YES	YES
18. Is posterior cruroplasty more effective than anterior cruroplasty in preventing de novo GERD following SG?	**Yes**	**Level II/III**	YES	NO	NO	YES
19. Does reducing the distance to the angle of His during sleeve gastrectomy increase the risk of de novo GERD?	**Yes**	**Level II**	YES	YES	YES (63%) NO (38%)	YES
20. Does antral resection during SG increase the incidence of de novo GERD compared to antral preservation?	**No**	**Level I**	YES	NO	NO (64%) YES (36%)	YES
21. Does avoiding functional stenosis at the incisura angularis and sleeve twisting help prevent GERD following SG?	**Yes**	**Level IV**	YES	YES	YES	YES
22. Does the use of a narrower bougie during sleeve gastrectomy increase the incidence of de novo GERD?	**No**	**Level I**,** II**,** III**	YES	YES	NO	YES
23. Is an alimentary limb of more than 50 cm a prevention for alkaline reflux in RYGB?	**Yes**	**Level I**	YES	NO	YES	YES
24. Is there a correlation between the size of the hiatal hernia and the incidence of GERD after OAGB?	**Yes**	**Level III**	YES	YES	YES	YES
25. Is a pouch length of > 10 cm a prevention of GERD in OAGB?	**Yes**	**Level II/III**	YES	NO	YES	NO

This approach allowed for an objective evaluation of the alignment between AI-generated inference and evidence-based human reasoning.

## Results

The responses provided by the different large language models (LLMs) demonstrated variability, both among themselves and in comparison, to the evidence-based inferences made by experienced surgeons. Notably, while some statements yielded consistent answers across models, others revealed significant discrepancies. Percentage agreement represents the proportion of the 25 statements for which the model’s Yes/No answer was identical to the predefined evidence-based expert response. Among the LLMs evaluated, ChatGPT-4o and Grok 3 each showed an 80% agreement with the evidence-based responses of surgeons. Gemini and Consensus.app both demonstrated a 72% level of agreement. A detailed summary of the comparative results is provided in Table 2.

## Discussion

Gastroesophageal reflux disease (GERD) and its associated complications remain significant clinical concerns following metabolic and bariatric surgery (MBS), particularly after procedures such as SG and OAGB [3, 9]. Both persistent and de novo GERD represent leading indications for revisional or conversional surgeries after primary bariatric procedures [10].

Although outcomes related to the prevention of de novo GERD vary across studies, there is consensus that optimal patient selection, MBS procedure selection, and precise technical aspects of surgical procedures are all critical factors in minimizing this risk.

In this evolving clinical landscape, artificial intelligence (AI), including large language models (LLMs), can serve as valuable adjuncts to human expertise in medicine, including MBS [8]. By synthesizing vast amounts of evidence-based data, AI can support clinicians in making more informed, personalized decisions aimed at reducing the incidence of de novo GERD following MBS. Integrating AI into surgical planning and postoperative care pathways has the potential to enhance risk stratification, optimize procedural choices, and ultimately improve patient outcomes. AI based responses presented a surprising high accordance to the answers provided by experts, up to 80% (ChatGPT and Grok 3.0). The patterns of responses were linear, because Authors decided to set a binary reply (yes/no), after a very punctual question.

1. **Can the surgeons rely only on GERD symptoms for definitive diagnosis of GERD before metabolic and bariatric surgery (MBS)?**
  In this comparative evaluation of four LLMs, all systems provided the same primary response to a binary (Yes/No) question, indicating consensus at the initial decision level. According to a clinical expert review, the information provided by the AIs was deemed suitable for patient education and as a basic reference for healthcare professionals beginning to explore the topic. Current clinical consensus underscores that symptoms alone are insufficient for diagnosing GERD. As established by the Lyon Consensus 2.0 [6], a definitive diagnosis requires objective evidence through tests such as upper endoscopy and pH monitoring, either wireless or catheter-based with impedance, supplemented by high-resolution manometry. Importantly, in patients with obesity, clinical symptoms are often unreliable predictors of GERD. Studies show that up to 40% of individuals with obesity may have asymptomatic (silent) GERD, with similar levels of acid exposure and reflux episodes regardless of symptom presence [11]. Additionally, mucosal hyposensitivity to acid may further obscure the clinical presentation in this population, complicating diagnosis and management [6].
2. **Is the current definition of GERD based only on symptoms?**
  A uniform ‘No’ response was given by three LLMs. Consensus.app highlighted, that GERD is based on the presence of troublesome symptoms and/or complications, not solely on symptoms. “The Montreal definition” in 2006 [12] defined GERD as the reflux of stomach contents into the esophagus, causing troublesome symptoms and/or complications. More recently, in 2024, the “Lyon 2.0 consensus” updated this definition, as not all ‘troublesome’ symptoms can be directly linked to reflux of gastric content, and symptoms alone are often insufficient for a conclusive diagnosis [6]. Thus, the currently valid modern definition of actionable GERD requires conclusive evidence of reflux-related pathology on endoscopy and/or abnormal reflux monitoring along with compatible troublesome symptoms.
3. **Are Upper Endoscopy and pH-monitoring necessary testing for GERD diagnosis before MBS?**
  This statement aimed to deepen the understanding of the role of instrumental testing in diagnosing GERD, particularly in the context of MBS. Three out of four LLMs responded with a “Yes” when asked whether objective testing is necessary for GERD diagnosis, aligning with current clinical guidelines and available evidence [6, 13, 14]. Gemini, however, answered “No,” arguing that diagnostic testing is not strictly required for all patients and should be based on individual clinical evaluation and surgical context.
4. **Is Esophageal High-Resolution Manometry mandatory for GERD diagnosis before MBS?**
  All four LLMs responded consistently with a “No” to this statement. Based on current evidence, high-resolution manometry (HRM) is considered an essential diagnostic tool for evaluating esophageal motility disorders, particularly in patients with non-obstructive dysphagia [14]. While HRM is not a primary diagnostic modality for GERD, it serves an important adjunctive role by providing supportive data [11]. Current clinical guidelines increasingly recognize HRM as a valuable component of the broader diagnostic approach to GERD, especially when used alongside pH monitoring and endoscopy to clarify ambiguous cases or guide management decisions.
5. **Are normative data for instrumental testing the same for the population with obesity?**
  Although all four LLMs consistently responded with a “No” to this statement, recent studies by Le et al. [15] and Tolone et al. [7] have provided new insights by establishing normative values for HRM and pH-impedance monitoring in patients awaiting MBS. These findings revealed slight differences compared to lean control groups. However, it is important to note that these datasets have not yet been widely adopted in routine clinical practice.
6. **Is a risk assessment for de novo GERD or worsening of existing GERD following MBS necessary during the preoperative evaluation for MBS?**
  A risk assessment for postbariatric GERD is crucial pre- and even intraoperatively, as previously undiagnosed hiatal hernias are recommended to be treated concomitantly at the index procedure [16–19]. All four LLMs agreed with the need for risk assessment, which can be done using the combination of objective and subjective GERD features, as described above. Accordingly, some patients may be more suitable to undergo RYGB over SG, or in case of SG, a hiatal repair with or without gastro- or phrenopexy can be planned upfront.
7. **Can the American Foregut Society (AFS) classification serve as a supplementary tool in identifying patients at risk for GERD following MBS?**
  The American Foregut Society (AFS) classification is a new endoscopic classification system for objective assessment of the esophagogastric junction integrity, which supposedly serves as a more comprehensive, standardized, and reliable method compared to the Hill system. It also accounts for the anatomical disruption of the diaphragmatic hiatus, including the diameter of the hiatal orifice and the axial extent of hiatal herniation. In a study in prebariatric patients, esophagitis rates increased stepwise and significantly with AFS grades, validating this classification for clinical practice [17]. This approach may be valuable in cases without available preoperative pH monitoring due to the time and/or cost reasons. All LLMs embraced this approach.
8. **Do patients with significant preoperative GERD have a higher risk for future GERD-related sleeve gastrectomy complications?**
  There is corroborative evidence stemming from cohort studies, national registries to support the observation that patients with preoperative GERD who undergo SG are at higher risk of having persisting or worsening symptoms [20]. Consequently, according to the USA national registry between 2015 and 2021, GERD remained the most common reason for conversion of SG, and RYGB was the most common conversion procedure [21]. Cohort studies using objective pre- and postoperative pH testing concluded that for patients with even mild symptoms, but positive pH testing (expressed by the DeMeester score), RYGB may represent a more durable surgical option [4]. The SM-BOSS RCT, which randomized patients without major GERD at baseline to SG or RYGB, found that at 10 years, 32.3% of SG patients reported de novo reflux symptoms, whereas this was only the case in 7.9% of the RYGB patients [22]. This has been answered correctly by all the LLMs.
9. **Is it reasonable to perform SG in patients who are well informed of the risk of worsening GERD requiring additional surgical interventions?**
  All four LLMs agreed with this approach. It is good practice to include in the prebariatric informed consent process, that patients undergoing SG are at higher risk of developing GERD as those having RYGB, further, that intractable GERD may require a conversion to RYGB in the future. Additionally, patients should also know, that based on intraoperative decision making, a hiatal hernia may be repaired concomitantly with the SG [19, 23, 24]. With this approach the risk of persisting or de novo reflux can be kept at bay and in case of severe GERD [23], they are better prepared to undergo conversional RYGB [18].
10. **Should SG be contraindicated in patients with silent GERD + large hiatal hernia?**
  While ChatGPT, Gemini, and Grok responded “YES” to this statement, Consensus.app replied “NO”. Emerging evidence, including a study by Lewis et al. [25], suggests that SG performed in combination with hiatal hernia (HH) repair results in greater excess weight loss (%EWL), improved GERD symptoms, and higher patient satisfaction compared to SG alone at least in the short term. This indicates that the addition of HH repair may mitigate GERD-related complications, challenging the notion that SG is inherently contraindicated in patients with hiatal hernias. A recent systematic review [26] emphasized that unrepaired HH is a clear risk factor for de novo or worsening GERD following SG, while repairing the hernia during surgery may substantially reduce this risk, as supported then by further studies [27–29].
11. **Is RYGB superior to SG for silent GERD patients, even without erosive esophagitis?**
  Recent meta-analyses and long-term studies consistently support the superiority of RYGB over SG in preventing de novo and worsening GERD, including in patients with silent GERD, those with abnormal reflux on pH-impedance testing but without symptoms or erosive esophagitis [30] and showed that RYGB reduces the risk of de novo GERD by approximately 70% compared to SG. Five-year follow-up data reveal higher GERD remission rates following RYGB. Notably, up to 30% of SG patients may require long term PPI and revisional surgery for refractory GERD, compared to less than 5% in RYGB recipients [30–33]. Moreover, asymptomatic GERD patients without visible mucosal damage may still experience severe reflux symptoms post-SG, while RYGB often serves as a therapeutic anti-reflux operation [34, 35]. When queried on whether RYGB is superior to SG for managing silent GERD, all four LLMs responded affirmatively, aligning with current high-level clinical evidence.
12. **Is RYGB superior to SG in against the occurrence of de novo GERD?**
  In response to this statement, all four LLMs unanimously affirm that RYGB offers superior protection. This consensus aligns with the current body of clinical evidence, which consistently demonstrates that RYGB’s anatomical reconstruction, including the bypass of the acid-producing stomach and duodenum, provides significant anti-reflux benefits [22, 30, 36]. In contrast, SG can exacerbate or induce GERD due to increased intragastric pressure and potential disruption of the lower esophageal sphincter. The LLMs correctly identify these mechanisms, although their responses vary in depth and may omit detailed risk stratification or patient selection considerations.
13. **Does SG have a higher risk for developing Barrett’s Esophagus and reflux esophagitis compared to RYGB?**
  All LLMs responded “yes” to this statement. SG has been increasingly associated with a higher risk of GERD and Barrett’s esophagus (BE) when compared to RYGB [37, 38]. Supporting this concern, a recent meta-analysis involving over 2,000 patients reported significant increases in postoperative symptomatic GERD, endoscopic evidence of esophagitis, and new-onset hiatal hernia. Most notably, it identified a 5.6% incidence of de novo BE, which tended to increase with longer-term follow-up [39].These findings underscore the importance of careful patient selection, preoperative evaluation for reflux risk, and ongoing surveillance in SG recipients, particularly in those with predisposing factors for GERD or BE.
14. **Is RYGB superior to OAGB against the occurrence of de novo GERD?**
  The responses from various LLMs to this statement are broadly aligned with current clinical evidence. All four models affirm that RYGB is generally more effective in reducing the incidence of de novo GERD compared to OAGB, although the Consensus.app provided a split response, indicating equal support for both ‘yes’ and ‘no’ regarding this statement. This consensus reflects available data showing that RYGB’s anatomical configuration, including the creation of a Roux limb, effectively minimizes both acid and bile reflux, whereas OAGB’s loop configuration is associated with a higher risk of bile reflux into the gastric pouch and esophagus [1, 40]. While the LLMs responses vary slightly in detail and explanation, they consistently support the conclusion found in current literature.
15. **Does OAGB increase GERD risk compared to RYGB in silent GERD patients?**
  Recent high-quality evidence indicates that OAGB carries a significantly greater risk of de novo GERD compared to RYGB, as confirmed by the responses of four LLMs. A recent meta-analysis by Kapellas et al. [1] reported that the odds of developing de novo GERD after OAGB were nearly six times higher than after RYGB. Further studies confirm these results, with GERD symptoms developing in up to 20% of OAGB patients, compared to less than 5% among RYGB patients [41, 42]. However, some studies suggest that remission rates for both preoperative and de novo GERD may be comparable between OAGB and RYGB in selected patients [43, 44]. These might be the reason, why consensus.app has a higher “No” rate (75%) in its answer.
16. **Can a small hiatal hernia (less than 2–4 cm) be left unrepaired during SG without increasing the risk of de novo GERD?**
  When evaluating the impact of small hiatal hernia (HH) repair during SG on the development of de novo GERD, responses from four LLMs revealed conflicting views. Notably, only ChatGPT-4 suggested that repairing small HHs might reduce the risk of de novo GERD. In contrast, Gemini, Consensus.app, and Grok 3 aligned with current evidence, indicating that leaving a small HH unrepaired does not increase the risk of postoperative GERD. This statement is supported by clinical data. Two studies suggest that routine repair of small hiatal hernias during SG may not significantly reduce the risk of de novo GERD. In a retrospective study by Ying Lye et al., 74% of patients developed GERD within six months post-SG, with no significant association between small, unrepaired HHs and GERD development [45]. Similarly, a randomized trial by Snyder et al. found no difference in GERD symptom improvement between SG patients with and without crural repair in patients with small HH [46]. These findings indicate that small HHs (≤ 4 cm) may not require routine repair, and GERD prevention should instead focus on individual symptom profiles and tailored surgical decisions.
17. **Does repairing a medium or large hiatal hernia during SG reduce the incidence of de novo GERD?**
  All four LLMs reviewed confirmed that repairing medium to large HHs during SG reduces the incidence of de novo GERD. This is broadly supported by clinical literature, though results across studies vary in magnitude and interpretation [16, 47–49]. Also, a meta-analysis by Chen et al. (2021) confirmed that SG with HHR significantly improves GERD symptoms and esophagitis rates. However, it also showed no statistically significant difference in de novo GERD incidence when comparing SG + HHR to SG alone, highlighting the limited impact of HHR on preventing new-onset GERD [50].
18. **Is posterior cruroplasty more effective than anterior cruroplasty in preventing de novo GERD following SG?**
  Current evidence indicates that posterior cruroplasty is more effective than anterior cruroplasty in reducing the risk of de novo GERD after SG. Among four LLMs queried on this topic, only ChatGPT-4 and Grok accurately reflected this evidence. A large retrospective study by Hider et al. involving 4,015 SG patients showed that posterior repairs led to significantly better outcomes, greater symptom improvement (69.5% vs. 64.0%), lower de novo GERD incidence (12.3% vs. 17.1% for anterior repair), and fewer complications such as hemorrhage and readmissions [16]. Similarly, El Chaar et al. found a trend toward higher satisfaction and better outcomes with posterior repairs, particularly when mesh was used, though differences were not statistically significant [27]. The anatomical advantage of posterior cruroplasty, allowing a tension-free, more robust closure of the diaphragmatic hiatus that may better protect the gastroesophageal junction, explaining its superior efficacy.
19. **Does reducing the distance to the angle of His during SG increase the risk of de novo GERD?**
  In response to this statement regarding whether reducing the distance to the angle of His during SG increases the risk of de novo GERD, all four LLMs consistently affirm this association. Their answers align with the current surgical literature, which indicates that a more aggressive resection near the angle of His may compromise the integrity of the gastroesophageal junction and the angle’s natural anti-reflux barrier [51]. This disruption, combined with increased intragastric pressure from a tighter sleeve, can predispose patients to the development of GERD postoperatively. The LLMs accurately reflect these biomechanical concerns, though with varying levels of explanation. Overall, their responses are concordant with the evidence, emphasizing the importance of surgical technique in minimizing postoperative reflux complications.
20. **Does antral resection during SG increase the incidence of de novo GERD compared to antral preservation?**
  Among the four LLMs, only ChatGPT-4 and Grok supported the association that antral resection during SG increases the incidence of de novo GERD compared to antral preservation. In contrast, Gemini and Consensus.app did not confirm this relationship. However, based on current Level I evidence, there appears to be no significant association between antral resection and an increased risk of de novo GERD. A randomized controlled trial by Singh et al. demonstrated that antral resection resulted in superior weight loss outcomes without a corresponding rise in reflux symptoms, as assessed at the 12-month postoperative follow-up [52]. Additionally, a comprehensive meta-analysis by Luo et al., which included 14 randomized controlled trials comprising a total of 1,222 patients, found no significant difference in surgical duration or the incidence of de novo GERD between the antral resection and antral preservation approaches in SG [53].
21. **Does avoiding functional stenosis at the incisura angularis and sleeve twisting help prevent GERD following SG?**
  This statement is supported by all studies and all 4 LLMs, although scientific literature offers only level IV evidence. Gastric twisting after SG is mainly associated with symptoms that include early satiety, epigastric pain associated with food intake, gastroesophageal reflux and early vomiting [54]. To classify gastric twist, a 3-stage classification was proposed by Siqueira et al., although a statistically significant correlation of twisting degrees was not observed for both the presence of symptoms and the degrees of esophagitis [55]. Furthermore, the SG morphology (torsion, stenosis at the incisura angularis, kinking, and fundus dilation) can be strongly related to post-operative GERD [56–58].
22. **Does the use of a narrower bougie during sleeve gastrectomy increase the incidence of de novo GERD?**
  Multiple pre- and postoperative modifications have been proposed to reduce GERD after SG, yet their effectiveness remains uncertain because the condition is multifactorial and its precise pathophysiology is still unclear [51]. In the largest cohort analyzed to date including 7,435 patients, a smaller bougie size coupled with a resection line placed farther from the pylorus was independently linked to a higher incidence of GERD two years after surgery, a relationship likely mediated by delayed gastric emptying and increased intraluminal pressure [59]. Conversely, a meta-analysis by Wang et al. demonstrated that using narrower bougies in SG resulted in more effective weight loss without increasing the risk of overall complications including GERD compared to larger diameter bougies [60]. Of the four LLMs consulted, three aligned with this interpretation, while the Consensus.app did not.
23. **Is an alimentary limb of more than 50 cm a prevention for alkaline reflux in RYGB?**
  This statement is supported by two key studies that emphasize the role of an alimentary limb longer than 50 cm in preventing alkaline (bile) reflux in RYGB (Level of evidence I), as showed in patients after total gastrectomy [61] and after revisional surgery for symptomatic bile reflux after RYGB [62].
  Grok 3, ChatGPT, and Consensus.app support the positive consensus, while Gemini answers negatively, stating that a longer alimentary limb in RYGB is crucial for preventing alkaline reflux, a specific length of more than 50 cm is not a guaranteed prevention, and optimal length can vary. This negative statement is not supported by specific evidence and might only be a different type of interpretation of scientific results. Gemini underlines that an alimentary limb is crucial, but does not support the specific length of 50 cm.
24. **Is there a correlation between the size of the hiatal hernia and the incidence of GERD after OAGB?**
  All LMMs have confirmed a correlation between the size of a HH and the incidence of GERD following OAGB. However, it is important to note that this association is not yet supported by high-grade evidence. The current level of evidence remains at Level III, as exemplified by a retrospective study. In that study, moderate to large hiatal hernias were identified as significant contributing factors for the development of de novo GERD following OAGB [5]. Based on these findings, the authors hypothesized that intraoperative identification and concurrent repair of a hiatal hernia during OAGB may be crucial for minimizing postoperative GERD-like symptoms and improving long-term patient outcomes.
25. **Is a pouch length of > 10 cm a prevention of GERD in OAGB?**
  This statement is supported by three different studies in the literature (Level of evidence II/III). According to the originators of the OAGB, due to patients’ morphological parameter variability, a standard length for the gastric pouch is not predefined. It must, however, begin below the crow’s foot [63, 64], going up to the angle of His. A multi-institutional survey from Musella et al. found a statistical correlation for postoperative duodenal-gastro-esophageal reflux with a preexisting GERD or with a gastric pouch shorter than 9 cm (*p* < 0.001 and *p* = 0.001), respectively. In their series, the length of the gastric pouch was 14.2 ± 3.4 cm (8.2–18.7-cm range) [65]. ChatGPT adopt the statement and Consensus.app agrees with the human work and emphasize correctly that these studies suggest that a pouch length of greater than 10 cm in OAGB may help reduce the risk of GERD, as shorter pouches (less than 9 cm) are associated with higher bile reflux, but GERD can still occur due to other factors and is not completely prevented by pouch length alone. Grok3 and Gemini does not agree and highlight that a pouch length of greater than 10 cm in OAGB is not a definitive prevention of GERD (Gemini) and that bile reflux risk depends more on anastomosis and limb length (Grok3).

## Limitations and Strengths

This study had several limitations. One key limitation was that not all statements were supported by high-level evidence; in some cases, conflicting results were observed across different studies. As a result, expert responses were based either on the highest available level of evidence or, when limited, on the only existing evidence. Another limitation was the restricted use of LLMs. We only evaluated four LLMs due to limited access to others. Also, we used Consensus.app as an LLM-based search engine and research assistant, not a standalone LLM, that uses AI to locate relevant scientific papers and then extracts and synthesizes findings from them, rather than generating answers solely from a single foundational model.

**Fig. 1:**
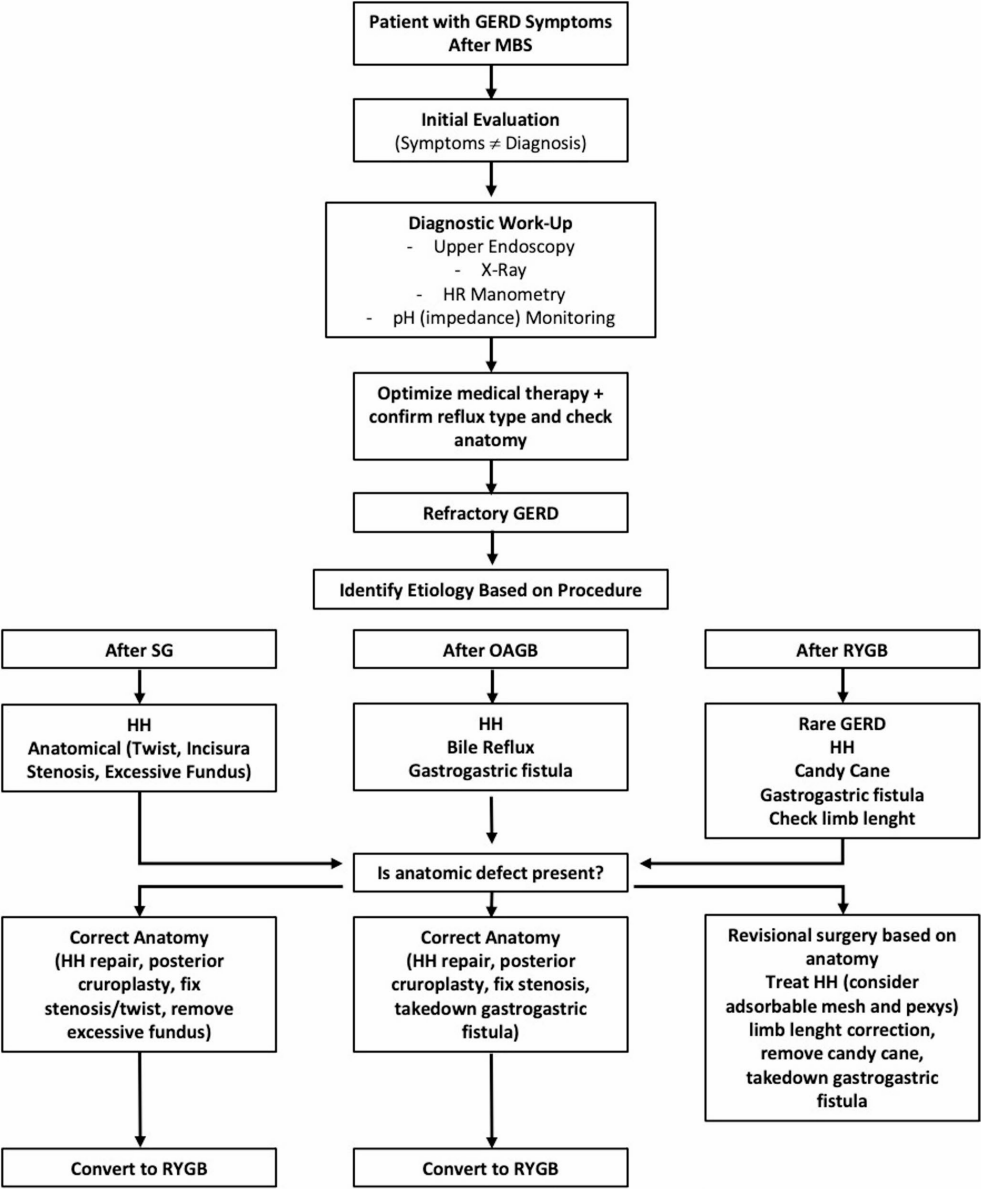
Proposed flowchart for the management of post operative GastroEsophageal Reflux Disease (GERD). MBS: Metabolic and Bariatric Surgery; HR: high resolution; SG: Sleeve Gastrectomy; RYGB: Roux-En-Y Gastric Bypass; OAGB: One Anastomosis Gastric Bypass; HH: Hiatal Hernia

Despite these limitations, the study has notable strengths. It comprehensively examined definitions, diagnostic methods, and all relevant factors influencing the prevention of de novo GERD. Importantly, this is the first study to integrate scientific evidence, expert judgment, and AI to address this topic holistically. A proposed flowchart derived by this integration is depicted in Fig. 1.

## Conclusion

De novo GERD is a relatively frequent complication following MBS, yet it remains largely preventable. Early identification of at-risk individuals through appropriate diagnostic tools, especially for asymptomatic GERD, is essential. Preventive strategies should also include careful patient selection, choosing the optimal surgical approach, and utilizing refined intraoperative techniques to minimize the risk of de novo GERD.

In this context, the use of AI, including LLMs alongside humans’ clinical expertise, offers a promising avenue to support surgical decision-making. AI can aid in analyzing complex datasets, identifying risk patterns, and guiding evidence-based strategies. When combined with a surgeon’s experience and judgment, AI has the potential to enhance clinical outcomes and reduce the incidence of de novo GERD after MBS.

## Data Availability

No datasets were generated or analysed during the current study.
